# Synergistic investigation of natural and synthetic C1-trophic microorganisms to foster a circular carbon economy

**DOI:** 10.1038/s41467-023-42166-w

**Published:** 2023-10-21

**Authors:** Enrico Orsi, Pablo Ivan Nikel, Lars Keld Nielsen, Stefano Donati

**Affiliations:** 1 The Novo Nordisk Foundation Center for Biosustainability, Technical University of Denmark, 2800 Kgs. Lyngby, Denmark; 2 Australian Institute for Bioengineering and Nanotechnology (AIBN), The University of Queensland, 4072 Brisbane, QLD Australia

**Keywords:** Metabolic engineering, Synthetic biology, Applied microbiology

## Abstract

A true circular carbon economy must upgrade waste greenhouse gases. C1-based biomanufacturing is an attractive solution, in which one carbon (C1) molecules (e.g. CO_2_, formate, methanol, etc.) are converted by microbial cell factories into value-added goods (i.e. food, feed, and chemicals). To render C1-based biomanufacturing cost-competitive, we must adapt microbial metabolism to perform chemical conversions at high rates and yields. To this end, the biotechnology community has undertaken two (seemingly opposing) paths: optimizing natural C1-trophic microorganisms versus engineering synthetic C1-assimilation de novo in model microorganisms. Here, we pose how these approaches can instead create synergies for strengthening the competitiveness of C1-based biomanufacturing as a whole.

## Abstract (web summary)

Using one carbon (C1) molecules as primary feedstock for bioproduction holds great potential for a circular and carbon neutral economy. Here, the authors discuss the potential of merging knowledge gained from natural and synthetic C1-trophic organisms to expedite the development of efficient C1-based biomanufacturing.

## Introduction

The development of a circular, bio-based economy is one of the grand challenges of this century. In fact, the use of biology for manufacturing (henceforth referred to as biomanufacturing) can be instrumental towards mitigating greenhouse-gas emissions into the atmosphere while supporting economic growth^1–3^. Biomanufacturing is historically rooted in bioprocesses using polytrophic organisms^2,4^, meaning heterotrophic organisms that grow on multicarbon feedstocks^5^. CO_2_ is fixed by crops into biomass, which is treated to release plant sugars for their biological conversion into value-added products^4^ by microbial cell factories^6^.

Although plants can successfully fix CO_2_ at ambient concentrations, they display a low energy conversion efficiency. It has been estimated that only 4.6–6% of sunlight energy is captured in biomass in C3 and C4 plants, respectively^7,8^. Moreover, empirical measurements of such conversions report that efficiencies can get lower than 3%^9,10^. Another drawback of plant-based production is the broader negative impact on planetary boundaries, including nitrogen- and phosphate-cycles, land-system change, fresh water use and the use of xenobiotics for pest control^11^. Efficient use of lignocellulosic residues is complicated by recalcitrance, which imposes the use of further inputs in energy and materials to extract plant sugars^12,13^. In addition, easily accessible sugars (e.g. starch, sucrose) directly compete with the food supply chain. Altogether, these characteristics render biomanufacturing fueled by plant-biomass uncompelling for a circular carbon economy capable of mitigating greenhouse gases emissions while limiting species loss, land and water use, and the introduction of xenobiotics in the ecosystems.

The use of photosynthetic microorganisms (e.g. cyanobacteria or microalgae) for biomanufacturing is also being explored extensively^14–16^. This is justified by several factors: being unicellular organisms, their cellular biomass is photosynthetically active; they present a faster growth-rate; do not require scarce resources such as arable land or freshwater; can be harvested throughout the whole year^16^. Therefore, they could be used as cell factories for the synthesis of value-added compounds. However, the reported values of photosynthetic efficiency for these species in pilot-scale reactors are in the order of 1.8–4.2%^17,18^. This, combined with major challenges in scaling up photosynthetic cultivations (i.e. contamination of open systems, low light availability along the optical path which require extensive land use) render the industrial application of photosynthetic microorganisms inadequate for large-scale biomanufacturing^19–21^.

**Fig. 1:**
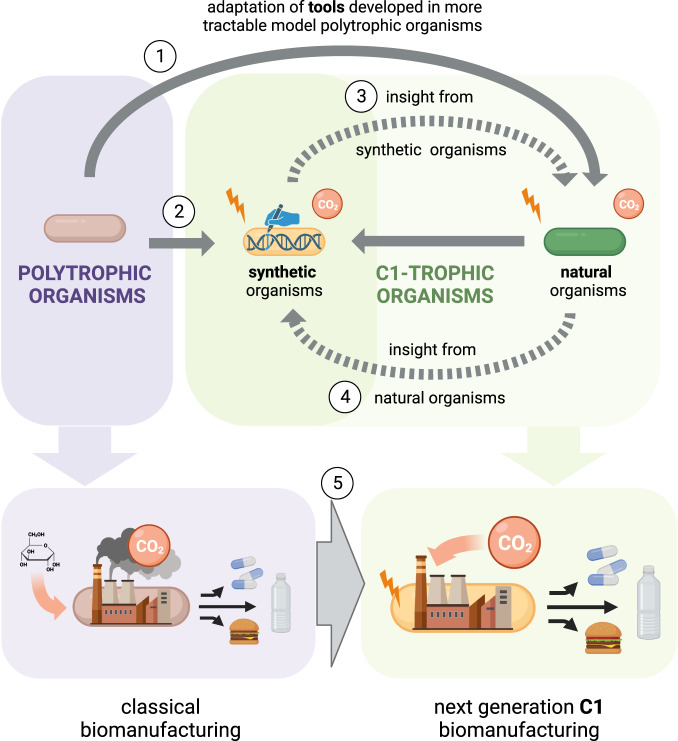
Overview of the current research in the field of microbial C1 assimilation. Systems and synthetic biology tools are usually developed in model polytrophic organisms, and are then applied to non-model organisms (1). Recently, some polytrophic organisms have been engineered to grow on C1 carbon sources (2). We argue that insight generated from such synthetic systems can help to improve natural C1-trophic organisms (3), which in turn should be studied to guide further engineering approaches (4). A next-generation of cell factories engineered to assimilate C1 feedstock could enable the switch from classical biomanufacturing to C1-based biomanufacturing (5). Created using BioRender.com.

**Fig. 2:**
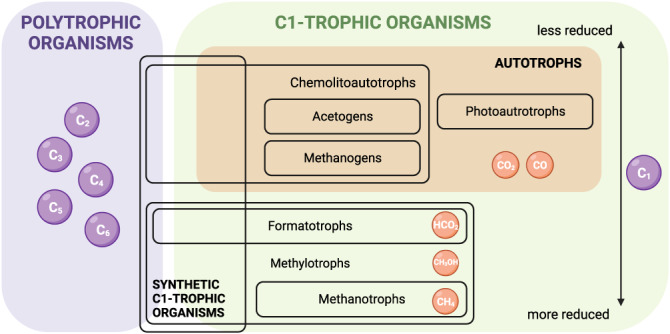
Landscape of polytrophic and C1-trophic organisms. C1 lifestyles are ordered by the level of reduction of the C1 compounds assimilated. At the intersection between polytrophic organisms and C1-trophic organisms lie the recently constructed synthetic C1-trophic microorganisms. Created using BioRender.com.

An alternative approach can rely on the use of photovoltaic solar cell modules to capture solar energy with reported conversion efficiencies for this technology reaching values of about 24%^22,23^. Electricity is then used to generate biologically available electron donors that can be used in microbial fermentations. The hydrogen evolution reaction can generate hydrogen (H_2_) from water with high selectivity and H_2_ can be used as an energy source in fermentations with CO_2_ as the carbon source. Alternatively, CO_2_ itself can be electrochemically upgraded into other more reduced C1-feedstocks^3,24,25^ (Fig. 1). These molecules (e.g. carbon monoxide, formate, methanol, and methane) can be generated with different efficiencies^25–29^ and can serve as both carbon and energy source. A variety of different microorganisms can then assimilate these C1 feedstocks (Fig. 2). In this article, we will refer to organisms able to grow on one-carbon compounds as C1-trophic organisms, as previously defined^30^.

Different levels of technological readiness (TRL) exist for each feedstock, with the highest values of TRL 9 reported for the electrochemical synthesis of H_2_ and a TRL 7–9 for the microbial conversion of C1-feedstocks like methanol, methane, and syngas^28^. These approaches are already superior to natural photosynthesis in terms of solar-to-product energy conversion^31–34^. Consequently, a plethora of studies exists in literature that describe the conversion of C1-feedstocks to value-added compounds via microbial cell factories^35–37^, with the first bioprocesses recently reaching industrial scale^38^.

Currently, most of the cell factories used for C1-based biomanufacturing are non-model microorganisms (Fig. 2). Their physiology is less understood than polytrophs such as e.g., *Escherichia coli* and *Saccharomyces cerevisiae*. Moreover, such non-traditional species present a less developed genetic toolkit than model polytrophs^39–42^. Because of these limitations of non-model species, a complementary approach has emerged in the past decade, which consists of rewiring the core metabolism of polytrophs to accommodate a C1-trophic behavior by means of synthetic biology. Illustrative examples will be discussed in a separate section in more detail and include, e.g., the implementation of the Calvin-Benson-Bassham (CBB) cycle^43^, the reductive glycine pathway (rGlyP)^44,45^, the ribulose monophosphate (RuMP) cycle^46,47^, and the serine threonine cycle (STC)^48^ in the model bacterium *E. coli*.

Current domains of synthetic biology and metabolic engineering for C1-feedstocks seemingly follow two distinct routes. On one hand, natural C1-trophic organisms are being studied and engineered to become more standardized and superior cell factories^49–54^; on the other hand, polytrophs which present well characterized metabolism and for which we possess advanced genome editing toolkits are being rewired to grow on C1-feedstocks by hefty synthetic biology endeavors^36^. This distinction has been highlighted in a recent, timely opinion article which contrasts natural with synthetic trophic modes (in the context of methanol assimilation), highlighting the inferior growth rates and product yields of synthetic methylotrophs^55^.

Although the two approaches seem to diverge or even compete against each other, we believe that they can contribute synergistically to the advancement of C1-based biomanufacturing (Fig. 1). In this perspective article, we aim to highlight the possible connections existing between the investigation of natural and synthetic organisms that are capable of using C1-feedstocks for growth. By using the schematic presented in Fig. 1, we define the links that already exist, and that we propose, between model and non-model organisms as well as natural and synthetic C1-trophic organisms. Each of these links are addressed in a dedicated section. Altogether, we reason that the fields of systems and synthetic biology can support this overarching goal for pushing forward C1-based biomanufacturing. We will also discuss how these links can advance our comprehension of C1 metabolism and its further optimization for a circular carbon economy.

## Systems and synthetic biology tools from model polytrophic to non-traditional C1-trophic organisms

**Fig. 3:**
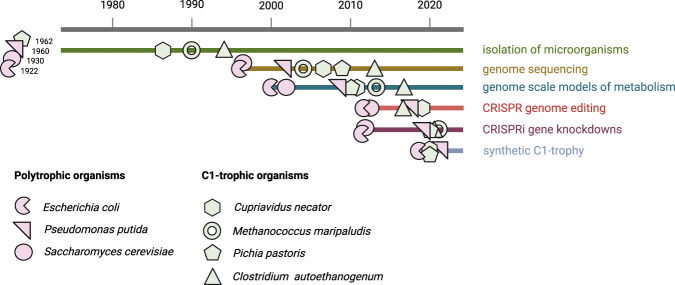
Timeline of development of systems and synthetic biology tools in selected polytrophic and C1-trophic organisms. Created using BioRender.com. Source data are provided as a Source Data file.

The historical development of systems and synthetic biology tools enabled a transition from methods exclusively available for model organisms to their expansion to non-canonical microbial species (Fig. 3). The advent of omics technologies in the 1990s and early 2000s enabled the systematic investigation of living systems as a whole, leading to the emergence of systems biology as a standalone discipline^56^. This has been facilitated by using model organisms such as *E. coli* and *S. cerevisiae*. In fact, as these species are abundant human commensals^57^, they have been used as models in biology since the first half of the 20th century^58^. Therefore, they represented ideal platforms around which to develop system biology tools, as our understanding of their genetics and physiology was already quite advanced.

The field of genetic engineering is considered to have started in the 1970s with the first recombinant DNA technologies. In particular, this decade was pivotal for reaching important milestones in molecular biology, such as the use of restriction enzymes, DNA recombination, and DNA sequencing^59^. The genome editing revolution brought on by CRISPR-based genetic engineering in the mid 2010s^60^ significantly accelerated the synthetic biology revolution. This breakthrough was also initiated in the two abovementioned model species, with first landmark works on the topic published in the early 2010s^61,62^. From that moment onwards, scientists have been able to investigate and engineer biological systems in an unprecedented way, expanding the use of CRISPR-Cas tools also to non-model species^39,40,63,64^ (Fig. 3).

Both *E. coli* and *S. cerevisiae* rely on a polytrophic lifestyle. Therefore, exploiting those organisms for method development has clear advantages, as it relies on energetic carbon feedstocks, which enable fast growth and hence increase the speed of technological development. Moreover, since the dawn of molecular biology, many genetic elements (e.g. plasmids, origin of replications, promoters, ribosome binding sites, etc.) have been characterized for these species. All this caused the establishment and validation of most new protocols and genetic engineering approaches using these organisms. Conversely, newly isolated microorganisms pose a challenge in terms of genetic manipulation and have much less characterized genetic parts. For this reason, the adaptation of novel systems and synthetic biology technologies has been lagging in non-model microorganisms, including C1-trophs. For example, only in the last few years toolboxes for genetic engineering were adapted for non-model C1-trophic organisms (Fig. 3). Nevertheless, although genome-editing tools of C1 assimilating microorganisms are still in their infancy, we can expect a significant increment in the amount of species domesticated through these tools in the years to come^40^.

## Synthetic C1-trophic metabolism

The technological advances highlighted in Fig. 3 have enabled the engineering of synthetic metabolisms at different scales^65^. Synthetic metabolism has been defined as the subfield of synthetic biology that attempts to engineer metabolic routes that do not exist in nature^66^. Here, we refer to synthetic metabolism as those synthetic biology and metabolic engineering efforts that rewire the metabolic network in an organism, often by introducing heterologous metabolic pathways or modules. In recent years, many efforts focused on engineering synthetic C1-trophy (e.g. autotrophy, formatotrophy or methylotrophy, Fig. 2), mimicking natural metabolic pathways that can assimilate C1, extensively reviewed elsewhere^36^.

In the case of the CBB cycle, two articles served as steppingstones towards the implementation of full synthetic autotrophy. The first milestone was reached in 2016 with the generation of a hemi-autotrophic *E. coli* strain synthesizing all its sugar phosphates through the CBB cycle^67^. Then, in 2018 the same cycle was functionally implemented in *Methylobacterium extorquens*, although it did not succeed in supporting full autotrophic growth^68^. The first complete demonstration of transforming a polytrophic organism into an autotroph was published in 2019, when *E. coli* was equipped with the CBB cycle to grow on CO_2_ and formate as sole carbon and energy sources^43^. In the same year, the methylotrophic yeast *Pichia pastoris* (now called *Komagataella pastoris*) was also converted into an autotrophic organism capable of growth on CO_2_ and methanol using the CBB cycle^69^. Apart from engineering synthetic autotrophy, in several studies non-methylotrophic heterotrophic organisms were transformed to attain a methylotrophic metabolic lifestyle. The reductive glycine pathway (rGlyP), first theorized as a synthetic pathway^70^ and then discovered to be natural^71^, was successfully implemented into *E. coli*^44,45^, in the knallgas bacterium *Cupriavidus necator*^72^, and in the soil bacterium *Pseudomonas putida* for growth on formate^73,74^. Anticipating the generation of a fully functional rGlyP, stepwise approaches with the validation of modular segments of the route were described for *E. coli* and *S. cerevisiae*^75–77^. Moreover, the rGlyP has been shown to also support methanol assimilation^45,74^. Similarly, the ribulose monophosphate (RuMP) cycle was implemented in *E. coli* for growth on methanol^46,47^. Also in the case of the RuMP cycle, several preliminary works, which did not realize a complete autocatalytic cycle, achieved important milestones towards that goal^78–81^. Altogether, these works provided valuable insight regarding the plasticity of microbial metabolism and demonstrated our ability to control and rewire metabolic networks.

Another approach to engineering synthetic metabolisms, is to design and implement completely synthetic pathways. Rather than relying on the metabolic repertoire present in nature^70^, this approach enables the creation of much more efficient routes to assimilate C1. A handful of new-to-nature synthetic CO_2_ fixation pathways have been created and tested in vitro in the last year. The first of such works included the crotonyl-CoA/ethylmalonyl-CoA/hydroxybutyryl-CoA (CETCH) cycle^82^. Then, the reductive Glyxoxylate and Pyruvate Synthesis (rGPS) cycle and the Malyl-CoA-Glycerate (MCG) pathway were combined into the rGPS-MCG cycle, which was shown to reach CO_2_ rates up to 0.55 mM/h^83^. Recently, the HydrOxyPropionyl-CoA/Acrylyl-CoA (HOPAC) cycle was also functionally assembled in vitro, converting about 3.0 mM of CO_2_ within two hours^84^. A synthetic variant of the serine cycle, the serine-threonine cycle, was first proposed by Arren Bar-Even^85^ and then implemented by his group in *E. coli*, demonstrating a new synthetic formate assimilation route in vivo^48^.

As a result of these engineering campaigns, our overall understanding of synthetic C1 assimilation is improving. The mutations responsible for autotrophy in *E. coli* were recently identified, and a strain with such (surprisingly few) mutations grew with comparable growth rates^86^. In the synthetic methylotrophic *E. coli*, only the expression of three heterologous genes and deletion of two genes were required to induce methylotrophy^47^ albeit at low growth rates, which could be improved after an adaptive laboratory evolution experiment of 250 generations.

Despite these successful attempts, the engineering of synthetic C1-trophic strains is still in its infancy. All strains constructed so far have lower growth rates and yields than their natural counterparts. For example, the evolved methylotrophic *E. coli* strain has a doubling time of 8 h^47^, which is still far from the 3 h doubling time of the natural methylotroph *Methylobacillus flagellatus*^87^. While *C. necator* can naturally grow on formate (through formate oxidation and the CBB cycle) with a doubling time of 3 h^88^, the fastest synthetic formatotrophic strain to date has a doubling time of 6 h^89^. Hence, there is significant room for improvement before synthetic C1 assimilating strains can be exploited for C1-based biomanufacturing.

## Understanding C1 metabolism through synthetic metabolism approaches

In the previous section, we presented the achievements in terms of synthetic C1-trophic routes within model microorganisms. Thanks to their better-characterized metabolism and genetic accessibility, polytrophs are ideal platforms in which to understand metabolic design principles that can be applied to the optimization of one-carbon metabolisms. In this section, we convey the idea that knowledge obtained from synthetic metabolism endeavors can facilitate our understanding, and consequent optimization, of metabolism and physiology in natural C1-trophic organisms. We provide four complementary examples supporting this thesis focused on investigating design principles of autocatalytic cycles, identifying possible evolutionary trajectories of carbon concentrating mechanisms, surpassing biomass yield in the natural CBB cycle via the rGlyP, and enhancing the thermodynamic drive of bioproduction pathways.

Most of the C1-assimilation pathways described in literature rely on autocatalytic cycles, namely pathways where one of the products is a catalyst for the pathway itself. The CBB cycle is an example of autocatalytic route because for every five molecules of ribulose 1,5-BP it consumes, it generates six ribulose 1,5-BP while fixing five CO_2_ molecules. To better understand the autocatalytic nature of the CBB cycle, a fundamental study of the dynamics and stability of autocatalytic cycles corroborated the importance of changes in substrate affinity of enzymes at the cycle’s branch points^90^. This evidence was confirmed in a further investigation, where the genetic basis of *E. coli*’s adaptation to a functional CBB cycle -described in the previous section^43,67^- was assessed in further detail through retro-engineering^91^. Mutation in three genes (*prs*, *serA*, and *pgi*) prevented efflux from the cycle towards biomass precursors. Moreover, modifications at two global regulators (*crp* and *ppsR*) proved to be essential for the transition from polytrophy to autotrophy. In a follow-up study looking at different lineages of evolved autotrophic strains, only 3 mutations (*pgi*, *crp* and *rpoB*) were identified and proved to be essential to enable autotrophy in *E. coli*^86^. The mutations were involved in preventing efflux from the CBB cycle (*pgi*) and increasing the redox potential (*crp*, *rpoB*). Introducing these mutations in a wild-type *E. coli* strain enabled autotrophy at similar rates to strains that were evolved for several generation^86^. By exploiting the naïve metabolic context of a polytrophic host, these findings provided mechanistic understanding for the implementation of a robust, autocatalytic CO_2_ fixation cycle.

In a similar fashion, *E. coli* was used as platform in which to reconstruct a bacterial CO_2_ concentrating mechanism (CCM)^92^. This study empirically demonstrated that ca. 20 genes from *Halothiobacillus neapolitanus* are required for successful CO_2_ fixation through Rubisco at ambient air condition. Together, these genes encode for an α-carboxysome structure capable of containing Rubisco and carbonic anhydrase as well as a transporter for inorganic carbon. *E. coli* harboring the α-carboxysomes from *H. neapolitanus* was further employed to determine the stoichiometry and the plasticity of such microcompartment^93^. The same carboxysome has been further employed in *E. coli* together with the expression of Rubisco activase components to enhance CO_2_ fixation^94^. Moreover, the *E. coli* strain harboring the complete CCM was used as control platform in which to study the paths of CCM evolution in autotrophic bacteria^95^. From a biotechnological perspective, a whole bacterial CCM has been heterologously expressed in autotrophic eukaryotes to enhance carbon fixation, e.g., by introducing synthetic α-carboxysomes within tobacco chloroplasts^96^.

The rGlyP is particularly remarkable as it is highly efficient in energetic terms, and it runs aerobically^70,97^. Even though the pathway was eventually discovered in nature, most studies around this route have focused on introducing it into polytrophic microorganisms. However, due to its high efficiency, its exploitation could be of interest also to improve growth in natural C1-trophic organisms. In fact, this was recently achieved by successfully implanting the pathway in *C. necator*^72^. Eventually, after further rounds of engineering and evolution, it has been postulated that such a synthetic strain could grow with higher yields (~15%) on formate compared to the wild type growing with the CBB cycle^98^. This evidence could be very impactful, suggesting that in the future relying on efficient synthetic C1 assimilation pathways might be of interest even in organisms naturally capable of growth on C1.

Principles of synthetic metabolism developed in polytrophs can also benefit bioproduction from C1 feedstocks. Of particular interest are those options that expand the design space for pathway optimization^99^. One example of this sort is represented by the malonyl-CoA bypass, which originates from *Streptomyces* sp. and was first realized in *E. coli* for the synthesis of mevalonate^100^. This bypass allows synthesis of acetoacetyl-CoA via the condensation of acetyl-CoA with malonyl-CoA (through combined activity of acetyl-CoA carboxylase and NphT7) instead of the condensation of two acetyl-CoA molecules (through acetoacetyl-CoA thiolase). Whereas the latter route is thermodynamically unfavorable (Δ_*r*_G’^m^ > 0 kJ/mol), the malonyl-CoA bypass results in a favorable reaction thanks to the investment of one ATP ((Δ_*r*_G’^m^ < 0 kJ/mol). In terms of bioproduction from C1 feedstocks, this bypass has been harnessed for enhancing photoautotrophic 1-butanol synthesis in *S. elongatus*^101^ and formate-dependent production of crotonate in *C. necator*^88^.

In summary, the studies presented in this section demonstrate how insight obtained in synthetic strains could be used to understand and improve C1 assimilation even in natural strains for biotechnological applications.

## Principles of natural C1-trophic organisms to guide the engineering of synthetic organisms

While studying synthetic organisms is a novel approach that can provide understanding into natural phenomena, insight from natural C1-trophic organisms has been (and still is) important for the engineering of synthetic metabolisms. Such organisms play a significant role in the planetary carbon cycle, and they have fine-tuned their metabolism over billions of years of evolution. We argue that modern quantitative systems biology studies of such natural non-model organisms can provide detailed blueprints to engineer synthetic strains. While many genome sequences were made available in the last decades and polytrophic microorganisms have been investigated thoroughly at the systems-scale, such studies of natural C1-trophic organisms are only starting to be performed.

For example, functional genomics studies have become fundamental to determine the function and essentiality of genes at a high throughput. Transposon insertion sequencing is a common method to verify the genotype–phenotype relationship of knockout mutants, which has been applied extensively in the last decade^102^. However, only in past few years this approach was applied to C1-trophic organism, more specifically on *C. necator*^103,104^ and *C. autoethanogenum*^105^. An alternative approach is by using pooled libraries of CRISPRi strains, in which the genotype-phenotype relationship can be investigated through knocking down gene expression over time^106–108^. In 2022, *Eubacterium limosum* was subjected to such a study, revealing the organism’s gene fitness essentiality in autotrophic and heterotrophic growth conditions^109^.

On the other hand, proteomics and metabolomics are powerful methodologies that can help us obtain quantitative understanding of how a cell is built and are particularly important for the investigation of metabolism. For example, it was shown that cells need to invest many resources in the expression of carbon assimilation enzymes. Accordingly, acetogens are known to devolve up to one third of their proteome to C1-trophic functions^110^. This high fraction of the proteome is not controlled tightly at the expression level, while fluxes are controlled at the thermodynamic level, by sensing changes to energy levels in the metabolome^111,112^. Similarly, the methanogen *M. maripaludis* utilizes up to one fourth of its proteome for methanogenesis^113^. Remarkably, the proteome maintains a constant allocation upon changes in growth rates, unlike for polytrophic microorganisms in which the anabolic and catabolic fractions of the proteome are regulated based on nutrient availability^114^. Also the facultative knallgas bacterium *C. necator* retains high expression levels of C1-trophic enzymes, even when grown on a multi-carbon source like fructose^103,115^. In fact, analyzing the metabolome and metabolic fluxes of *C. necator* also suggested that when grown on multi-carbon sources, the organism maintains a mixotrophic metabolic regime^116,117^, which appears to be an investment in readiness to quickly restore autotrophic growth^103^. Hence, it appears that natural microorganisms have evolved to maintain their proteome at high capacity for growth on C1 substrates, regardless of their presence in the environment. Understanding which other genes are regulated in different conditions might pinpoint unknown mechanisms by which natural strains maintain homeostasis. To this end, methodologies such as independent component analysis of transcriptome data might help reconstructing in detail the gene regulatory network, but have not been applied so far to natural C1-trophic organisms^118^. Combining reconstructed gene regulatory networks with quantitative metabolomics methods of C1 metabolism^119,120^ in different conditions could be used to pinpoint which metabolites are involved in mechanisms that change gene expression^121^ and in maintaining metabolic homeostasis^122^.

Given the high proteome fraction needed for C1-trophic growth, furthering our understanding of the regulation of enzymatic catalytic rates is of key importance. However, this is still a challenge even in microorganisms like *E. coli*, in which metabolite-protein interactions are widely unknown^123^. Novel systematic methodologies might help in the quest to unravel these interaction networks also in natural C1 assimilating strains^124,125^. For example, a recent study employed one of such systematic methods (LiP-SMap, limited proteolysis-small molecule mapping) proposing novel allosteric interactions for enzymes of the CBB cycle of photoautotrophs and of *C. necator*^126^. Another recent work shed light on the allosteric inhibition of the phosphoketolase XPK of Synechococcus elongatus PCC7942, revealing how the enzyme responds to low ATP levels to drain CBB cycle’s intermediates towards the formation of acetyl-phosphate^127^.

In summary, insight into how nature has shaped C1-trophic organisms can streamline the engineering of synthetic organisms, providing basic design principles. In fact, synthetic evolved strains often end up mimicking natural metabolic architectures. For example, when evolving synthetic methylotrophy in *E. coli*^47^, levels of the methanol dehydrogenase increased significantly (up to 40% of the proteome), and fluxes rearranged so that TCA anaplerosis would conserve carbon, through the PEP carboxylase or malic enzyme, similarly to what can be observed in natural methylotrophs^128^. Guiding engineering campaigns with insight into the architecture of natural C1 metabolism and its regulation can therefore help in speeding up the construction of robust synthetic strains.

## An outlook on next generation C1-based biomanufacturing

Although natural organisms have evolved to grow optimally on C1 in the environment, they are not immediately suitable for C1-based biomanufacturing. Such organisms might need to be adapted or engineered to become fermenterphiles, meaning microorganisms perfectly adapted to grow in the environment of a bioreactor^129^. In fact, there have been few successful cases of C1-based biomanufacturing at the industrial scale to date, which all relied on using natural C1-trophic strains, either adapted/evolved or genetically engineered. Natural methanotrophs are exploited to produce single-cell protein for animal feed (Calysta, calysta.com; Unibio, unibio.dk). More recently, engineered acetogenic bacteria were used to convert C1 compounds into C2 and C3 compounds in a carbon-negative fashion (Lanzatech)^38^.

On the other hand, the utilization of synthetic C1-trophic organisms is in its early days, as synthetic strains are often unfit for efficient growth and bioproduction. Despite some initial proof-of-concepts^89,130^, to the best of our knowledge, there are no cases of synthetic C1-trophic organisms used for large-scale biotechnological processes. Achieving an effective *plug&play* implantation of C1 assimilation in established cell factories will open the possibility of quickly testing it into different genetic backgrounds. This might be particularly important as different species might be innately more or less tolerant to high concentrations of C1 compounds^73,88,131^. As many polytrophic microorganisms have been dominating the biotech industry in the past, a wealth of information is available about their physiology and upscaling to industrial settings.

When feeding cells with C1 feedstocks, preventing resource waste (i.e. excessive oxidation of the substrate) will also become of key importance to render C1-based biomanufacturing economically competitive. Preventing resource waste will be fundamental to adopt C1-based biomanufacturing in life support systems used for space exploration applications, in which resources are limited and need to be recycled constantly^132–134^. In this context, the utilization of more efficient synthetic assimilation pathways should lead to higher yields. Evolving or engineering enzymes with higher catalytic rates might enable the construction of strains with a lower protein burden in catabolic enzymes, capable of more efficient growth^135^. Moreover, the utilization of minimal cell factories can play a big role in preventing resources diverting into useless portions of biomass. Model heterotrophic microorganisms have been widely engineered for this purpose^136^. On the other hand, genome reduction of natural C1 assimilating microorganisms is still lagging, with a single very promising recent achievement: in a recent study, it was demonstrated that a genome reduced strain of *C. necator* grows with higher growth rates and slightly higher yields^137^. Combining genome reduction approaches with synthetic assimilation pathways will eventually support efficient biomass or product formation from C1 feedstocks.

In the near future, it is likely that industrial processes will continue to rely on unmodified or engineered natural strains with purely synthetic strains quickly catching up. The choice of the initial organism will be influenced by regulatory frameworks, especially in places where rules for genetically engineered organisms are stricter (e.g. the European Union). In either case, combining the knowledge obtained in natural and synthetic strains will speed up the process of constructing more efficient strains and deliver a next generation of C1-trophic biofactories. Eventually, the adoption of such engineered strains will have to be considered along other challenges at the level of bioprocess engineering^129^ towards ultimately supporting efficient C1-based biomanufacturing.

Throughout this perspective article we propose a call to action for the communities working on natural- and synthetic-C1 trophic organisms to join their efforts in studying C1 metabolism as a whole. We believe that thanks to this approach the whole sector of C1-based biomanufacturing can increase its impact within the circular economy paradigm.

### Source data

Source Data
